# Cell recognitive bioadhesive‐based osteogenic barrier coating with localized delivery of bone morphogenetic protein‐2 for accelerated guided bone regeneration

**DOI:** 10.1002/btm2.10493

**Published:** 2023-01-18

**Authors:** Yun Kee Jo, Bong‐Hyuk Choi, Cong Zhou, Sang Ho Jun, Hyung Joon Cha

**Affiliations:** ^1^ Department of Biomedical Convergence Science and Technology School of Convergence, Kyungpook National University Daegu Republic of Korea; ^2^ Cell and Matrix Research Institute, Kyungpook National University Daegu South Korea; ^3^ Nature Gluetech Co., Ltd Seoul Republic of Korea; ^4^ School of Stomatology, Shandong University Jinan China; ^5^ Department of Oral and Maxillofacial Surgery Korea University Anam Hospital Seoul Republic of Korea; ^6^ Department of Chemical Engineering Pohang University of Science and Technology Pohang Republic of Korea

**Keywords:** barrier coating, bone morphogenetic protein‐2, guided bone regeneration, mussel adhesive proteins, titanium mesh

## Abstract

Titanium mesh (Ti‐mesh) for guided bone regeneration (GBR) approaches has been extensively considered to offer space maintenance in reconstructing the alveolar ridge within bone defects due to its superb mechanical properties and biocompatibility. However, soft tissue invasion across the pores of the Ti‐mesh and intrinsically limited bioactivity of the titanium substrates often hinder satisfactory clinical outcomes in GBR treatments. Here, a cell recognitive osteogenic barrier coating was proposed using a bioengineered mussel adhesive protein (MAP) fused with Alg–Gly–Asp (RGD) peptide to achieve highly accelerated bone regeneration. The fusion bioadhesive MAP‐RGD exhibited outstanding performance as a bioactive physical barrier that enabled effective cell occlusion and a prolonged, localized delivery of bone morphogenetic protein‐2 (BMP‐2). The MAP‐RGD@BMP‐2 coating promoted in vitro cellular behaviors and osteogenic commitments of mesenchymal stem cells (MSCs) via the synergistic crosstalk effects of the RGD peptide and BMP‐2 in a surface‐bound manner. The facile gluing of MAP‐RGD@BMP‐2 onto the Ti‐mesh led to a distinguishable acceleration of the in vivo formation of new bone in terms of quantity and maturity in a rat calvarial defect. Hence, our protein‐based cell recognitive osteogenic barrier coating can be an excellent therapeutic platform to improve the clinical predictability of GBR treatment.

## 1 INTRODUCTION

In dental implant therapy, the reconstruction of alveolar ridges with adequate levels of volume and quality is critical for the successful treatment of bone defects.^1^ Guided bone regeneration (GBR) is a widely used technique as part of the process for local augmentation and defect restitution in the alveolar bone, prior to dental implant placement.^2^ Barrier membranes for GBR prevent the invasion of rapidly proliferating nonosteogenic cells (i.e., epithelial and fibrous connective cells in the surrounding soft tissues) physically into the defect while maintaining space to repopulate slowly growing osteoprogenitor cells, subsequently guiding the formation of new bone.^3^, ^4^ As one of the most commonly used GBR membranes, titanium mesh (Ti‐mesh) provides excellent performance, particularly in enabling space support for osteogenesis and in adapting to various bone defects through bending and shaping due to its superior mechanical properties, and has good biocompatibility.^5^ However, the Ti‐mesh membrane is often susceptible to soft tissue intervention through the pores of the mesh.^6^ In addition, inadequate or sometimes delayed bone regeneration still occurs, mainly due to the limited bioactivity of inert titanium substrates.^7^, ^8^ Therefore, an advanced design of bioactive barrier membranes with regenerative ability is required to accomplish reliable clinical outcomes using the GBR approach.

To foster the surface properties and microenvironment favorable to rapid bone regeneration, several bioactive molecules, such as extracellular matrix (ECM) components and growth factors, have been added onto titanium surfaces to directly enhance the adhesion and differentiation of osteoprogenitors.^9^, ^10^ Among these, bone morphogenetic protein‐2 (BMP‐2), an osteoinductive growth factor that strongly facilitates the recruitment and differentiation of mesenchymal progenitors into mature osteoblasts, has been extensively utilized to functionalize the surface of substrates under the approval for safety and efficacy by the U.S. Food and Drug Administration (FDA).^11^, ^12^, ^13^ However, typical approaches of physical adsorption often involve an initial burst release of loaded BMP‐2 cargos with a low retention rate, which may lead to reduced therapeutic activity and potential adverse events associated with high‐dose or repeated administration (e.g., local inflammation, soft tissue edema, seroma, and unintended ectopic bone formation) in clinical settings.^12^, ^13^, ^14^, ^15^ In chemical immobilization strategies, covalent bonds impair the active sites of proteins and inevitably decrease their bioactivity.^16^, ^17^

**FIGURE 1:**
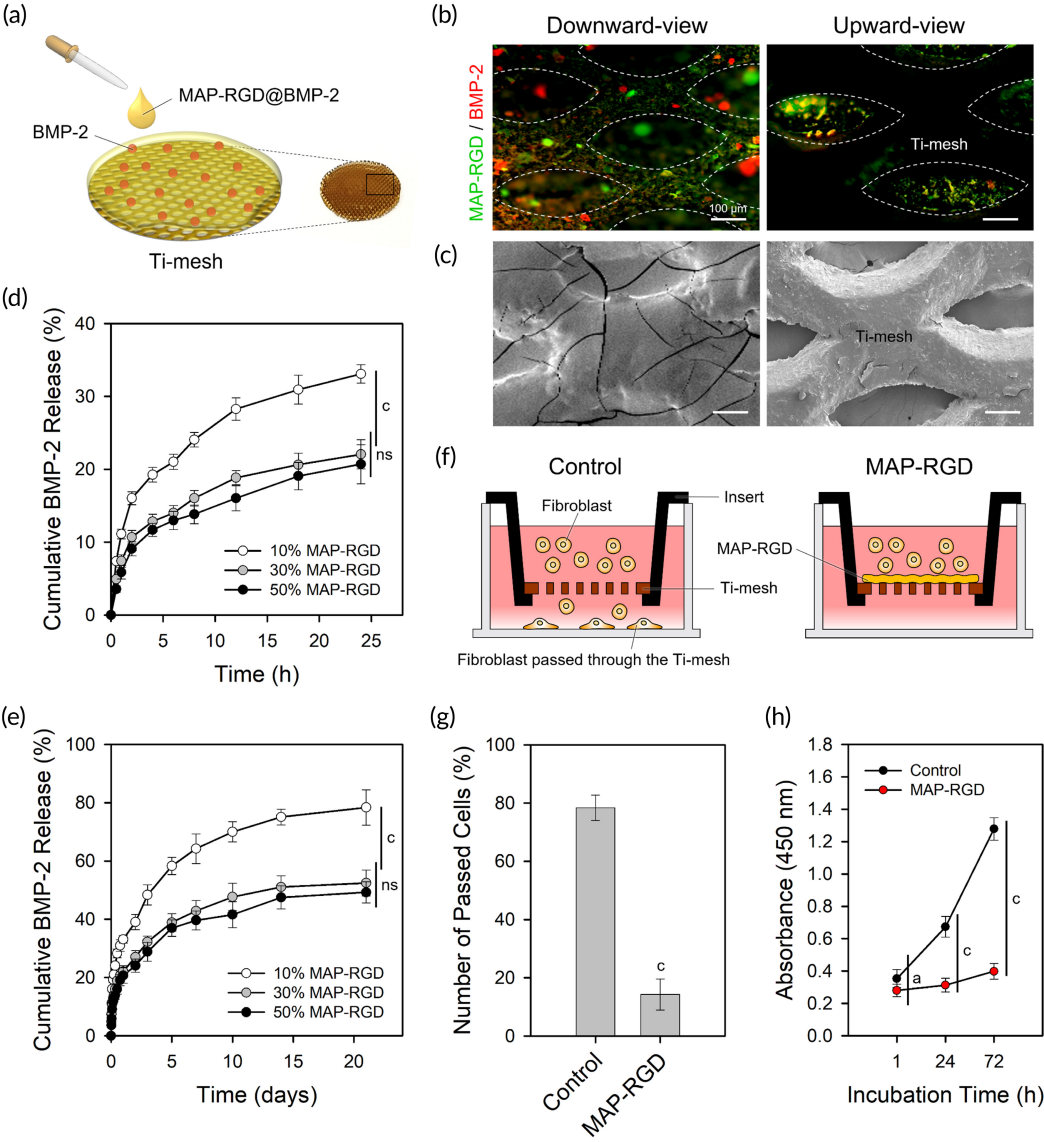
Preparation and characterization of the MAP‐RGD@BMP‐2 coating. (a) Schematic illustration of the preparation of the MAP‐RGD@BMP‐2‐coated surface. (b) Fluorescence and (c) SEM images of the MAP‐RGD@BMP‐2‐coated surface. For visualization, FITC‐labeled MAP‐RGD (MAP‐RGD^FL^) and RITC‐labeled BMP‐2 (BMP‐2^RHO^) were used. BMP‐2 release profiles from the MAP‐RGD@BMP‐2‐coated surface for (d) 24 h and (e) 3 weeks. (f) Schematic illustration of barrier permeability testing. (g) Number of cells that passed through the bare and MAP‐RGD‐coated Ti‐mesh surfaces after incubation for 24 h. (h) Cell counts on the bare and MAP‐RGD‐coated Ti‐mesh surfaces after incubation for 1, 24, and 72 h. The data are presented as the mean ± SD with statistical significance (one‐way ANOVA with Tukey's post hoc test). *p* < 0.05 and *p* < 0.005 are noted as “a” and “c,” respectively. Statistical insignificance is noted as “ns.” BMP, morphogenetic protein‐2; Control, bare Ti‐mesh surface; MAP, mussel adhesive protein; MAP‐RGD, MAP‐RGD‐coated Ti‐mesh surface; RGD, Alg–Gly–Asp.

In this work, we propose an osteogenic barrier coating capable of allowing osteoprogenitor cells to exclusively repopulate the bone defect site underlying the Ti‐mesh surface through a stepwise stimulation of bone regeneration utilizing a bioengineered protein glue that displays a surface‐decorated cell recognition motif and offers a sustained release of BMP‐2. Inspired by marine mussels, mussel adhesive proteins (MAPs) have increasingly emerged as promising bioglues with surface‐independent adhesion ability, biocompatibility, and biodegradability.^18^, ^19^, ^20^ We employed a bioengineered MAP fused with a cell recognitive Alg–Gly–Asp (RGD) peptide (MAP‐RGD) that facilitates osteogenic cellular behaviors by activating intracellular signaling pathways^21^ to serve as a passive barrier inside the pores of the Ti‐mesh and simultaneously functionalizing the surface of the titanium substrate (Figure 1a). In addition, positively charged MAP‐RGD can readily immobilize BMP‐2, which has a negative charge,^22^ enabling prolonged retention of BMP‐2 on the Ti‐mesh surface. Thus, we generated a MAP‐RGD‐based BMP‐2‐releasing osteogenic barrier coating (MAP‐RGD@BMP‐2) on the Ti‐mesh membrane, and evaluated its barrier performance and bone regenerative effects in vitro and in vivo.

## 2 RESULTS AND DISCUSSION

### 2.1 Construction of the MAP‐RGD@BMP‐2‐based osteogenic barrier coating

The simple gluing of a blended solution (MAP‐RGD@BMP‐2) of MAP‐RGD and rhBMP‐2 onto the Ti‐mesh membrane successfully generated a barrier coating layer that covered the pores of the Ti‐mesh and immobilized BMP‐2 (Figure 1b). The final BMP‐2 loading concentration in the MAP‐RGD@BMP‐2 coating was ~1.84 mg/ml (loading efficiency of 61.34 ± 0.36%), which is higher than the clinically used supraphysiological dose (usually 1.5 mg/ml) under approval of the U.S. FDA,^23^ and is suitable to study whether the MAP‐RGD can act as an effective carrier of BMP‐2 to overcome the high‐dose‐associated clinical risks and accomplish successful bone regeneration. From a microtopographical perspective, the MAP‐RGD@BMP‐2‐coated side of the surface (downward‐view) was slicker, whereas the opposite bare side of the Ti‐mesh (upward‐view) was relatively rougher (Figure 1c). The MAP‐RGD@BMP‐2 coating exhibited a typical burst release of BMP‐2 in the initial phase regardless of the concentration of MAP‐RGD, which corresponds to BMP‐2 being primarily located on the external surface of the coating layer (Figure 1d). Noticeably, a sustained release of BMP‐2 appeared on all the MAP‐RGD@BMP‐2 coated Ti‐mesh surfaces over 3 weeks (Figure 1e), primarily due to electrostatic interactions between the negatively charged BMP‐2 and the positively charged lysine residues rich in MAP‐RGD, as well as the intrinsic adhesive property of MAP‐RGD.^22^, ^24^ At 21 days postincubation, the Ti‐mesh surface coated with 10% (w/v) MAP‐RGD showed ~80% release of BMP‐2, while approximately half of BMP‐2 remained on the 30% (w/v) MAP‐RGD‐coated surface, which might be attributed to the stronger adhesive property of higher concentrations of MAP‐RGD. There was no more significant decrease in BMP‐2 release upon further increase in the MAP‐RGD concentration, indicating slight saturation of BMP‐2 binding by MAP‐RGD. Thus, we suggest the 30% MAP‐RGD coating is optimal for sustained release of BMP‐2 to accomplish successful outcomes in bone regeneration through an appropriate rate of biodegradation of the coated layer. These BMP‐2 release profiles showed a similar pattern to the degradation profiles of coating layer according to the concentration of MAP‐RGD; 10% (w/v) MAP‐RGD‐coated surface exhibited remarkably higher degradation ratio compared to 30% and 50% (w/v) MAP‐RGD‐coated surfaces (Figure S1, Supporting Information), which indicates a good correlation between BMP‐2 release and MAP‐RGD degradation. The ratios of BMP‐2 releases were higher than the ratios of coating degradation at all MAP‐RGD concentrations over the time periods, which can be attributed to a preferential release of BMP‐2 held at the outermost layer of coatings. Taken together, the sustained release of BMP‐2 loaded within the MAP‐RGD coating enabled durable local delivery of BMP‐2 in bone lesions, preventing risks related to short in vivo half‐life of BMP‐2 by systemic circulation^25^ as well as providing a better microenvironment for a prolonged period of time for bone healing.

To evaluate the soft tissue occlusive ability of the MAP‐RGD coating layer, the passage of fibroblasts across the MAP‐RGD‐coated Ti‐mesh membrane was analyzed using a customized transwell insert (Figure 1f). The MAP‐RGD coating layer effectively hindered the passage of cells through the Ti‐mesh membrane (Figure 1g,h). The leakage of cells (~14%) across the MAP‐RGD‐coated layer could be explained by the 20–30 μm of some holes generated by extracellular proteases secreted from fibroblasts at 1 day after seeding (Figure S2, Supporting Information).^26^ While cells that passed through the bare Ti‐mesh membrane exhibited normal proliferation, there was no significant proliferation below the MAP‐RGD‐coated Ti‐mesh membrane over 3 days because very few cells crossed the coating layer. Thus, the MAP‐RGD‐coated Ti‐mesh membrane can serve as an effective physical barrier to prevent soft tissue invasion into the bone defect and to maintain the space for effective bone regeneration.

### 2.2 In vitro osteogenic cellular behaviors on the MAP‐RGD@BMP‐2 coating layer

**FIGURE 2:**
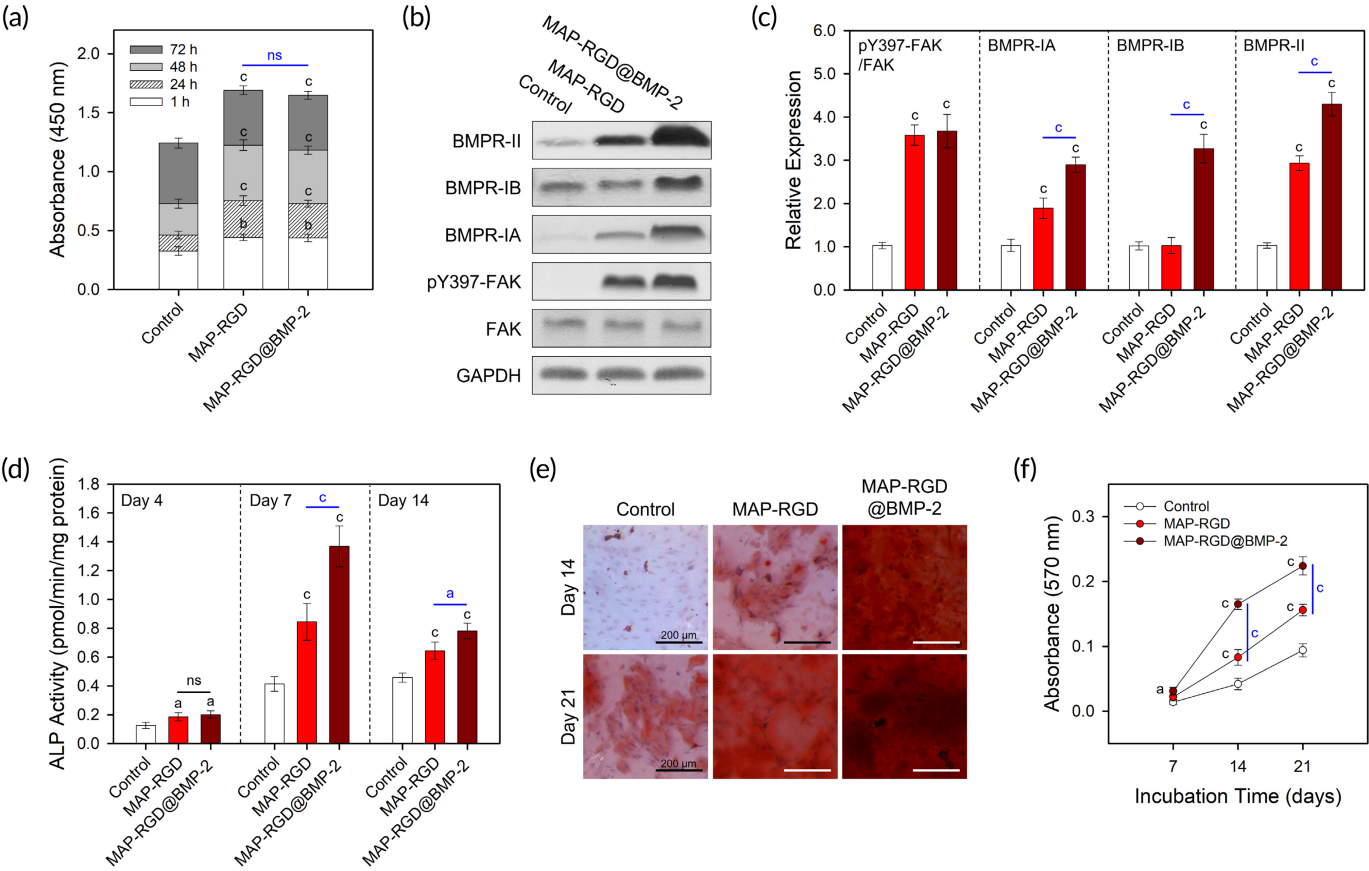
In vitro osteogenic behaviors of MSCs on the MAP‐RGD@BMP‐2‐coated surface. (a) Cell counts for the initial 3 days. (b) Western blot images of the protein expression of FAK, pY387‐FAK, BMPR‐IA, BMPR‐IB, and BMPR‐II in cells at 1 day after induction. (c) Relative quantitative levels of FAK phosphorylation and BMP‐2 receptor expression normalized to GAPDH. FAK activation was quantified through the relative band intensity of pY397‐FAK to FAK. (d) ALP activity at 4, 7, and 14 days after induction of differentiation. (e) Optical microscopic images and (f) calcium deposition levels of Alizarin Red S‐stained cells at 14 and 21 days after induction of differentiation. The data are presented as the mean ± SD with statistical significance (one‐way ANOVA with Tukey's post hoc test). *p* < 0.05 and *p* < 0.005 are noted as “a” and “c,” respectively. Statistical insignificance is noted as “ns.” BMP, morphogenetic protein‐2; Control, bare surface; MAP, mussel adhesive protein; MAP‐RGD, MAP‐RGD‐coated surface; MAP‐RGD@BMP‐2, MAP‐RGD@BMP‐2‐coated surface; RGD, Alg–Gly–Asp.

To verify the ability of the MAP‐RGD@BMP‐2 coating as a bioactive GBR barrier to facilitate bone regeneration, the osteogenic cellular behaviors of MSCs were systematically analyzed in vitro. MAP‐RGD@BMP‐2 was coated onto polystyrene surfaces to reduce the microtopographical variables of the Ti‐mesh surface, including surface roughness and microstructure. A bare polystyrene surface and a BMP‐2‐free MAP‐RGD‐coated surface were used as comparative controls. The number of attached MSCs at 1 h after seeding and their proliferation levels for the initial 3 days on the MAP‐RGD‐ and MAP‐RGD@BMP‐2‐coated surfaces were significantly higher than those on the bare surface (Figure 2a), mainly due to the intrinsic adhesive properties of MAP and the ability of the cell recognitive RGD motif to trigger integrin‐mediated signaling pathways.^21^, ^27^ Based on the similar level of cell counts on the MAP‐RGD‐ and MAP‐RGD@BMP‐2‐coated surfaces over the period of incubation, we expect that the MAP‐RGD coating can provide a highly osteoconductive microenvironment to support the retention and engraftment of osteogenic progenitor cells regardless of the existence of BMP‐2.

The expression levels of a phosphorylated focal adhesion kinase (FAK) in integrin‐mediated signaling pathways and BMP receptors (BMPRs), including type IA BMP receptor (BMPR‐IA), type IB BMP receptor (BMPR‐IB), and type II BMP receptor (BMPR‐II) were analyzed to explore the crosstalk between the cell recognitive RGD motif and osteoinductive BMP‐2 at the molecular level. From the immunoblotting results, MSCs on both the MAP‐RGD‐ and MAP‐RGD@BMP‐2‐coated surfaces exhibited a higher level of FAK phosphorylation compared to the bare surface, which was consistent with the cell proliferation results (Figure 2b,c). In general, in response to integrin‐binding, the signal transduction pathway is triggered through activation of FAK by phosphorylation of its tyrosine‐397 residue; this is followed by the organization of clusters with cytoskeletal proteins to connect actin filaments to transmembrane integrins.^7^, ^28^ Considering that FAK phosphorylation directly regulates the strength of cellular adhesion and several subsequent intracellular responses, such as proliferation and spreading,^7^, ^28^ the higher FAK phosphorylation on the MAP‐RGD‐ and MAP‐RGD@BMP‐2‐coated surfaces could be considered to be coupled with the higher rates of cell proliferation. In addition, we observed the highest upregulation of all three BMPRs in cells on the MAP‐RGD@BMP‐2‐coated surface. Similar to other members of the transforming growth factor‐β (TGF‐β) pathway, BMP‐2‐mediated signaling transduction pathways are triggered upon binding of BMP‐2 to the cell transmembrane serine/threonine kinase receptors BMPR‐I and BMPR‐II.^29^ We suggest that MAP‐RGD aids the interaction of surface‐bound BMP‐2 with BMPRs by providing the RGD motif as a cell adhesion site for substrates.^30^, ^31^ In addition, the MAP‐RGD‐coated surface exhibited higher expression of BMPR‐IA and BMPR‐II than the bare surface, confirming that RGD‐mediated integrin engagement stimulates intracellular cascades toward an osteogenic phenotype.^32^ Meanwhile, the nonsignificant difference in the BMPR‐IB expression between the bare surface and the MAP‐RGD‐coated surface implies that the combination of BMP‐2 with integrin binding ligands is crucial for the crosstalk of BMP‐2 receptors with integrins and synergistic osteogenic signaling.^33^, ^34^

To assess the osteopromotive ability of the MAP‐RGD@BMP‐2 coating, we utilized low concentrations of the osteogenic stimulants (corresponding to 10% of the commonly used concentration required for the induction of osteogenic differentiation) for the culture of MSCs. From the colorimetric assays, both the MAP‐RGD and MAP‐RGD@BMP‐2 coatings led to significant increases in alkaline phosphatase (ALP) activity at 4, 7, and 14 days after induction (Figure 2d). The MAP‐RGD@BMP‐2‐coated surfaces showed the highest level of ALP activity at 7 and 14 days. Given that ALP plays a key role in inorganic pyrophosphate metabolism in the maturation phase of osteogenic differentiation,^35^, ^36^ cells on the MAP‐RGD‐ and MAP‐RGD@BMP‐2‐coated surfaces seemed to be in intracellular processes prior to the stage of matrix deposition. Notably, the ALP activities of MSCs on both the MAP‐RGD‐ and MAP‐RGD@BMP‐2‐coated surfaces peaked after 7 days of culture and thereafter decreased, possibly due to transition into the mineralization phase. As expected, highly enhanced deposition of calcium minerals was detected on the MAP‐RGD‐ and MAP‐RGD@BMP‐2‐coated surfaces after 14 and 21 days of differentiation induction, as observed by Alizarin Red S staining (Figure 2e,f). The highest mineral deposition was observed on the MAP‐RGD@BMP‐2‐coated surface at each time point, which was consistent with the ALP activity results.

**FIGURE 3:**
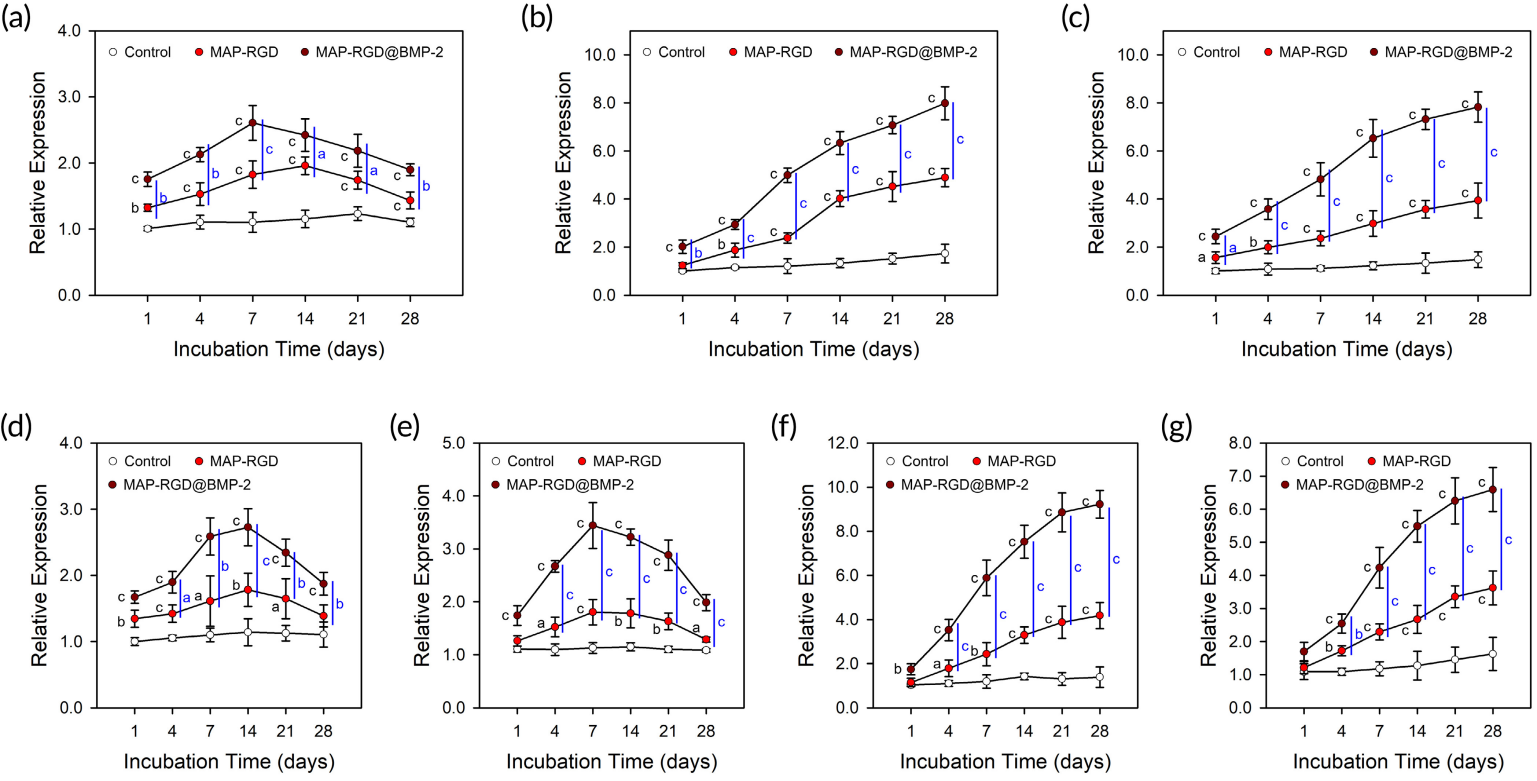
In vitro gene expression levels of osteoblastic lineage‐specific transcription factors, including (a) Runx2, (b) OSX, and (c) ATF4 at 1, 4, 7, 14, 21, and 28 days after induction of differentiation, and osteogenic markers, including (d) T1Col, (e) ALP, (f) BSP, and (g) OCN at 1, 4, 7, 14, 21, and 28 days after induction of differentiation. The data are presented as the mean ± SD with statistical significance (one‐way ANOVA with Tukey's post hoc test). *p* < 0.05, *p* < 0.01, and *p* < 0.005 are noted as “a,” “b,” and “c,” respectively. Control, bare surface; MAP, mussel adhesive protein; MAP‐RGD, MAP‐RGD‐coated surface; MAP‐RGD@BMP‐2, MAP‐RGD@BMP‐2‐coated surface; RGD, Alg–Gly–Asp.

The osteoblastic lineage‐specific commitment of MSCs on the MAP‐RGD@BMP‐2 coating was evaluated by quantifying the expression levels of several transcription factors and osteogenic markers using real‐time reverse transcription‐polymerase chain reaction (RT‐PCR). The expression levels of all transcription factors, including runt‐related transcription factor (Runx2), osterix (OSX) and activating transcription factor 4 (ATF4), that directly modulate the expression of genes required for osteogenic commitment and differentiation^35^, ^36^ were all notably upregulated in cells on the MAP‐RGD‐ and MAP‐RGD@BMP‐2‐coated surfaces throughout the entire culture period from 4 days after differentiation induction, exhibiting the highest upregulation on the MAP‐RGD@BMP‐2‐coated surface (Figure 3a–c). Runx2 is a crucial transcription factor that modulates the expression of osteogenesis‐related genes during the earliest phase of osteogenic differentiation and act as an upstream regulator of OSX expression,^37^ and ATF4 plays a role as a cofactor of Runx2.^38^ OSX contributes to the differentiation of Runx2‐expressing precursors into mature osteoblasts.^39^ The expression of Runx2 in cells on the MAP‐RGD@BMP‐2‐coated surface peaked at Day 7 postinduction and then decreased gradually, similar to previous studies on BMP‐2‐induced osteoinduction.^40^, ^41^, ^42^ Cells on the MAP‐RGD‐ and MAP‐RGD@BMP‐2‐coated surfaces exhibited gradual increases in the expression of OSX and ATF4 over the culture period of 28 days. Considering the peak of Runx2 expression in cells on the MAP‐RGD‐coated surface at Day 14 postinduction, the incorporation of BMP‐2 into MAP‐RGD coating could enable faster transition into subsequent stages of bone regeneration, including osteogenic maturation and mineral deposition.^42^

In addition, cells on the MAP‐RGD‐ and MAP‐RGD@BMP‐2‐coated surfaces showed significant upregulation of osteogenic markers, including type I collagen (T1Col), ALP, bone sialoprotein (BSP), and osteocalcin (OCN) from Days 4 to 28 postinduction (Figure 3d–g). We found the most remarkable upregulation of all osteogenic markers in cells on the MAP‐RGD@BMP‐2‐coated surface, even at Day 1 postinduction on the earliest phase of differentiation, which could be coupled with the subsequent upregulation of transcription factors and the eventually enhanced phenotypes of ALP activity and calcium deposition. Interestingly, the expression of T1Col in cells on the MAP‐RGD‐ and MAP‐RGD@BMP‐2‐coated surfaces exhibited peaks at Day 14 postinduction, implying cell aging with the extension of incubation time after matrix synthesis during differentiation.^35^, ^36^, ^43^ The ALP expression levels on the MAP‐RGD‐ and MAP‐RGD@BMP‐2‐coated surfaces with peaks at Day 7 postinduction are consistent with the Runx2 expression and ALP activity. These surfaces exhibited gradual increases in expressions of BSP and OCN, the primary markers of mineralization phase,^35^, ^36^ over the time periods. Collectively, we expect that the MAP‐RGD@BMP‐2 coating effectively generates a highly osteogenic microenvironment to osteoprogenitor cells on the underlying surface by enabling synergistic crosstalk between the RGD peptide and BMP‐2 in a surface‐bound manner.

### 2.3 In vivo bone regeneration on the MAP‐RGD@BMP‐2‐coated Ti‐mesh membrane

**FIGURE 4:**
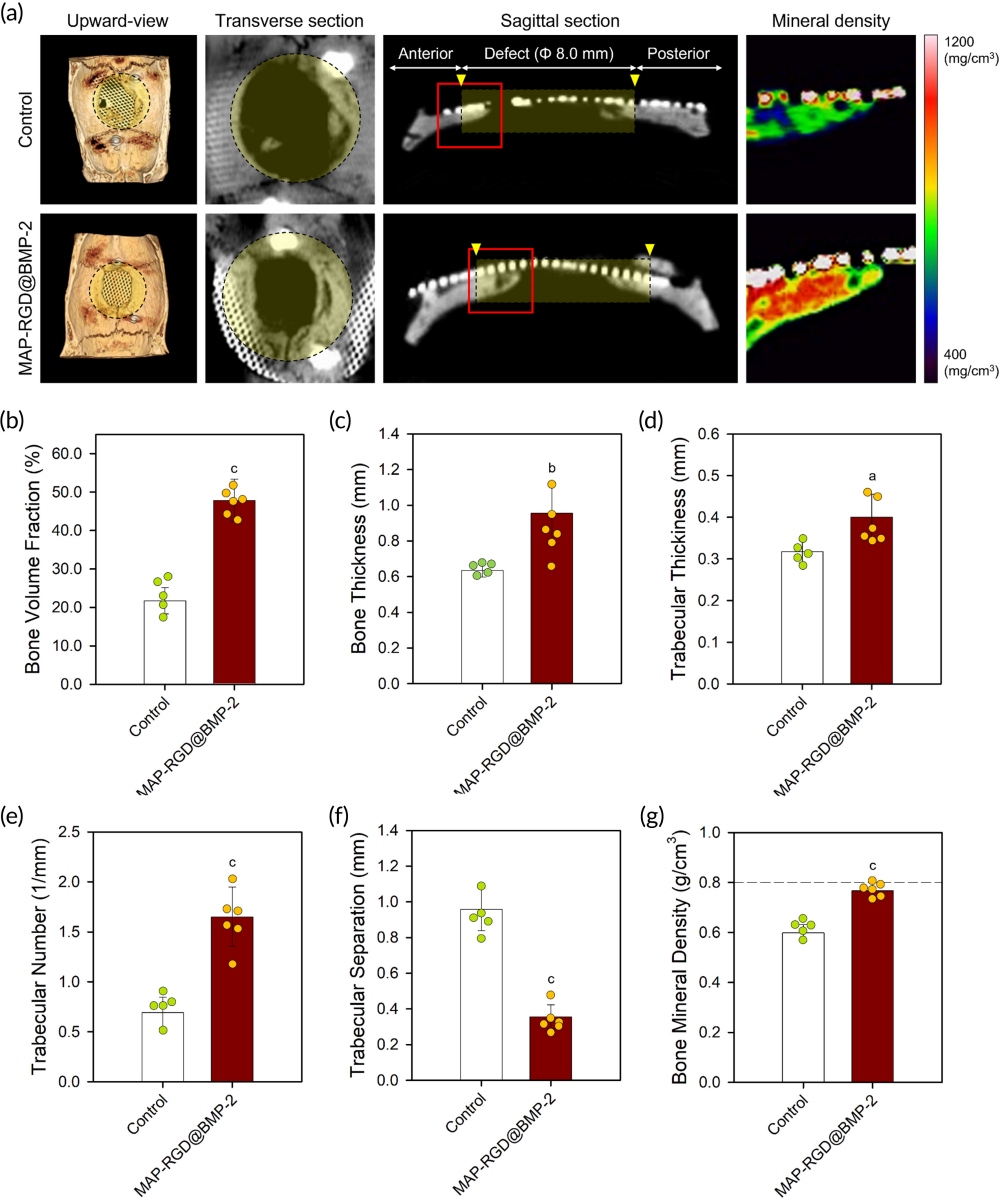
In vivo bone formation on the MAP‐RGD@BMP‐2‐coated Ti‐mesh surface in a rat calvarial defect model (*n* ≥ 5). (a) Microcomputed tomographic images with three‐dimensional (3D) rendering for an upward‐view, a transverse section, a sagittal section, and a mineral density coloration. The yellow areas indicate the defect site, and the yellow triangles indicate the marginal defect point. In the sagittal coloration for mineral density, the red color indicates relatively higher density, and the blue color indicates lower density. (b) Bone volume fraction, (c) bone thickness, (d) trabecular thickness, (e) trabecular number, (f) trabecular separation, and (g) bone mineral density of the defect site at 8 weeks after surgery. The data are presented as the mean ± SD with statistical significance (Kruskal–Wallis test with Dunn's post hoc test). *p* < 0.05, *p* < 0.01, and *p* < 0.005 are noted as “a,” “b,” and “c,” respectively. BMP, morphogenetic protein‐2; Control, bare surface; MAP, mussel adhesive protein; MAP‐RGD, MAP‐RGD‐coated surface; MAP‐RGD@BMP‐2, MAP‐RGD@BMP‐2‐coated Ti‐mesh surface; RGD, Alg–Gly–Asp.

To evaluate its ability to accelerate GBR in vivo, the MAP‐RGD@BMP‐2 coating, which had the most optimal bioactive features for enhancing osteogenic behaviors of MSCs in vitro, was applied onto the Ti‐mesh membrane and then implanted into critical‐sized rat calvarial defect site. A bare Ti‐mesh membrane was employed as a negative control. At 8 weeks after surgical implantation, the induction of new bone formation at the defect site was evaluated using three‐dimensional (3D) reconstructed μ‐CT images of the mineralized tissue. In bone rendering and cross‐sectional images, the defect of the MAP‐RGD@BMP‐2‐coated Ti‐mesh group was significantly recovered with newly formed bone (Figure 4a). A more than two‐fold increase in the volume fraction (%) of the defect filled with new bone in the MAP‐RGD@BMP‐2‐coated Ti‐mesh group (~48%) was observed compared to the bare Ti‐mesh group (~22%) (Figure 4b). The newly formed bone along the MAP‐RGD@BMP‐2‐coated Ti‐mesh membrane exhibited a significantly higher thickness than that on the bare Ti‐mesh membrane (Figure 4c). In addition, higher trabecular thickness (Tb.Th), higher trabecular number (Tb.N), and lower trabecular separation (Tb.Sp) were detected on the defect of the MAP‐RGD@BMP‐2‐coated Ti‐mesh group than on that of the bare Ti‐mesh group, indicating a more compact and well‐connected trabecular microstructure of the new bone (Figure 4d–f). Noticeably, the mineral density of bone formed on the MAP‐RGD@BMP‐2‐coated Ti‐mesh membrane was much higher than that on the bare Ti‐mesh membrane, and was comparable (~96%) to that of normal bone tissue (Figure 4g).

**FIGURE 5:**
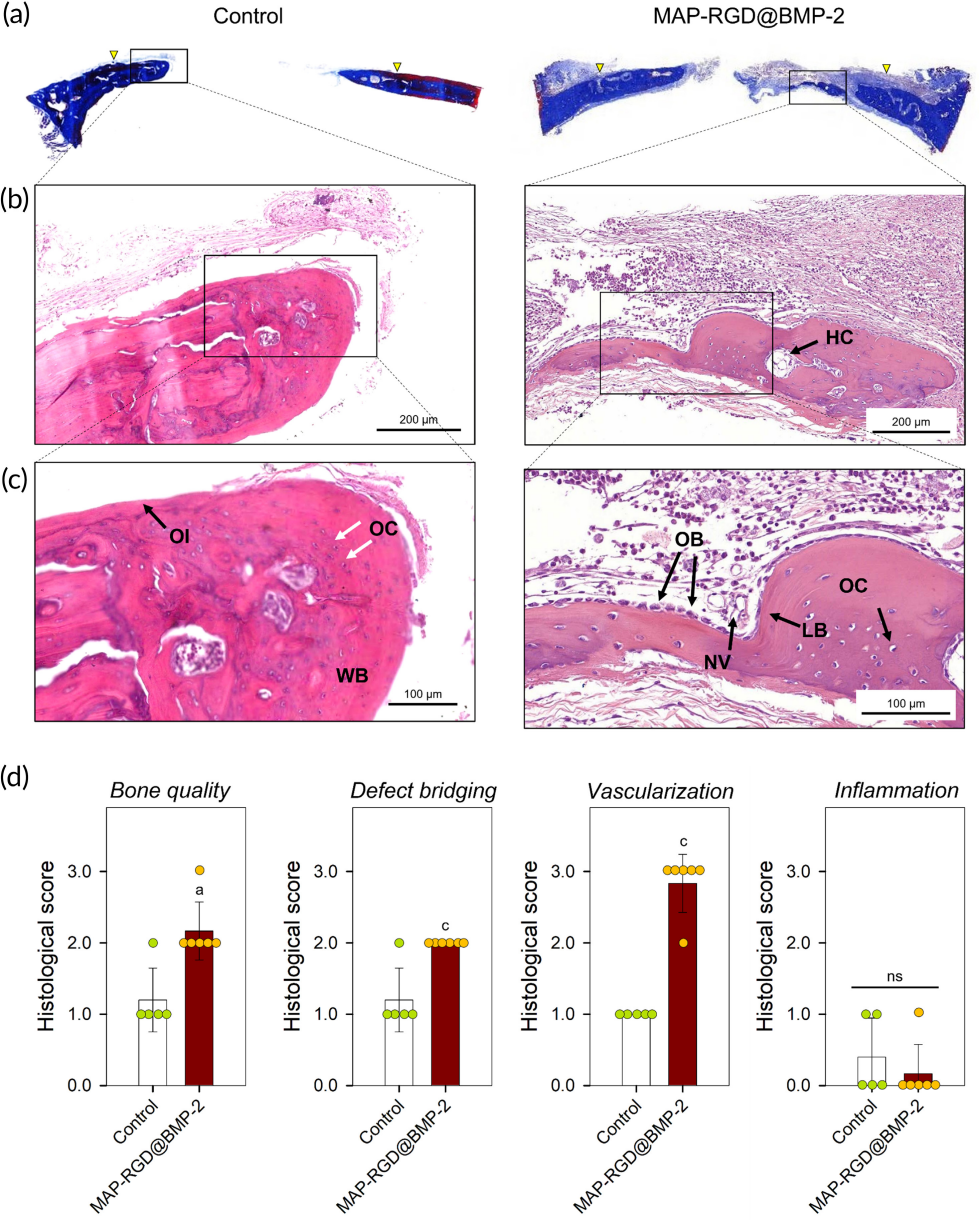
Histological analyses of newly formed bone on the MAP‐RGD@BMP‐2‐coated Ti‐mesh surface in a rat calvarial defect model (*n* ≥ 5). (a) Images of MT‐stained sections. The yellow triangles indicate the marginal defect point. (b,c) Images of H&E‐stained sections. (d) Semiquantitative histological scores. The data are presented as the mean ± SD with statistical significance (Kruskal–Wallis test with Dunn's post hoc test). *p* < 0.05 and *p* < 0.005 are noted as “a” and “c,” respectively. Statistical insignificance is noted as “ns.” Control, bare Ti‐mesh surface; HC, Haversian canal; H&E, hematoxylin and eosin; LB, lamellar bone; MAP, mussel adhesive protein; MAP‐RGD@BMP‐2, MAP‐RGD@BMP‐coated Ti‐mesh surface; Masson's trichrome; NV, new blood vessel; OC, osteocyte; OB, osteoblast; OI, osteoid; WB, woven bone; RGD, Alg–Gly–Asp.

We performed histological analyses by visualizing decalcified sections using Masson's trichrome (MT) and hematoxylin and eosin (H&E) staining to assess the morphology of newly regenerated bone tissue within the defect. In MT‐stained sections, a larger amount of bone tissue was newly formed along the Ti‐mesh membrane within the defect of the MAP‐RGD@BMP‐2 coating group than in the bare Ti‐mesh group (Figure 5a), consistent with the radiographic results. In the high‐resolution images of the H&E‐stained sections, maturely mineralized lamellae of the bone matrix with lacunae‐embedded osteocytes and Harversian canals were clearly found in the MAP‐RGD@BMP‐2‐coated Ti‐mesh group, whereas the bare Ti‐mesh group displayed a relatively immature structure of woven bone (Figure 5b–d). In particular, even though osteocytes were observed in both groups, the linings of mature osteoblasts at the rims of the bone matrix were found only in the MAP‐RGD@BMP‐2 coating group. There was no significant evidence of an adverse inflammatory response in both groups. Given that the MAP‐RGD@BMP‐2 coating showed an outstanding ability to form a large amount of highly matured bone in vivo, we suggest that it can be successfully employed to generate an osteogenic microenvironment on the GBR membrane and its use can be further expanded to various medical prostheses.

## 3 CONCLUSION

Herein, we propose a mussel protein‐based GBR barrier coating capable of facilely coating a Ti‐mesh membrane with a cell recognitive RGD motif and osteoinductive BMP‐2 to accelerate bone regeneration. The simple gluing of MAP‐RGD onto the Ti‐mesh membrane allowed the formation of a cell‐occlusive physical barrier to block the invasion of fibroblasts. The MAP‐RGD coating demonstrated potential as an effective osteogenic therapeutic platform through localized delivery and sustained release of BMP‐2. The synergistic crosstalk of the matrix‐bound RGD peptide and BMP‐2 enabled distinguishably enhanced osteogenic cellular behaviors in vitro and accelerated bone regeneration in a rat calvarial defect in vivo. Thus, our proposed MAP‐RGD@BMP‐2 osteogenic barrier coating can open new avenues to accomplish satisfactory clinical outcomes in bone therapies as a promising and practical GBR approach with further expansion to more general bone tissue engineering including titanium‐based prostheses.

## 4 MATERIALS AND METHODS

### 4.1 Preparation of MAP‐RGD@BMP‐2‐coated Ti‐mesh surface

MAP‐RGD which consists of the sequences of fp‐151 and C‐terminus GRGDSP was produced using *Escherichia coli* and was highly purified as previously described.^7^, ^21^, ^27^ The production and purification of MAP‐RGD were assessed by 12% (v/v) sodium dodecyl sulfate‐polyacrylamide gel electrophoresis (SDS‐PAGE). The concentration of protein was determined by the Bradford assay (Bio‐Rad, Hercules, CA, USA) with a standard of bovine serum albumin (BSA; Promega, Madison, WI, USA).

A Ti‐mesh membrane (Cytoplast Osteo‐Mesh; Osteogenics Biomedical, Lubbock, TX, USA) with a thickness of 0.2 mm and a pore size of 0.3 mm was cut into circular plates with a diameter of 9 mm. The prepared MAP‐RGD was dissolved in endotoxin‐free distilled water (DW) at a 30% (w/v) concentration. The Ti‐mesh surface was spread with 100 μl of a blended solution of MAP‐RGD and recombinant human BMP‐2 (rhBMP‐2; Daewoong, Seoul, Korea; 3 mg/ml) and incubated at 37°C overnight, following the previously established protocol^27^; then, it was washed with phosphate‐buffered saline (PBS; Sigma‐Aldrich, St. Louis, MO, USA) three times to remove excess MAP‐RGD. The BMP‐2 loading efficiency was calculated by measuring the amount of BMP‐2 washed out (i.e., excess and loosely tethered BMP‐2) after the washing process using a BMP‐2 enzyme‐linked immunosorbent assay (ELISA) kit (R&D Systems, Minneapolis, MN, USA) according to the manufacturer's protocol.

### 4.2 Characterization of MAP‐RGD@BMP‐2‐coated Ti‐mesh surface

The coating of MAP‐RGD@BMP‐2 on the Ti‐mesh surface was analyzed using a fluorescence microscope (Olympus, Tokyo, Japan). For visualization, fluorescein‐labeled MAP‐RGD (MAP‐RGD^FL^) and rhodamine‐labeled BMP‐2 (BMP‐2^RHO^) were prepared by reacting the fluorescence isothiocyanate (FITC) isomer (Sigma‐Aldrich) and the rhodamine isothiocyanate (RITC) isomer (Sigma‐Aldrich), respectively, with the proteins for 3 h in a 0.1 M sodium bicarbonate buffer (pH 9.0). The unreacted dyes were removed via dialysis with a 6000–8000 molecular weight cutoff (MWCO) membrane (Spectrum, New Brunswick, NJ, USA). The surface morphology of the Ti‐mesh membrane after the coating procedure was observed using scanning electron microscopy (SEM; JSM‐6010LV; JEOL, Tokyo, Japan) after gold sputtering.

To investigate the BMP‐2 release behavior, the MAP‐RGD@BMP‐2‐coated surfaces were prepared using 10%, 30% or 50% (w/v) MAP‐RGD and were incubated in PBS under shaking at 37°C. Each solution was sampled at predetermined time points, and the removed volume was replaced with an equivalent volume of fresh PBS buffer. The BMP‐2 released in each buffer was measured using a BMP‐2 ELISA kit (R&D Systems). The degradation of MAP‐RGD coatings was analyzed by measuring the amount of released MAP‐RGD protein via Bradford assay with a protein standard of BSA. The MAP‐RGD‐coated surfaces were incubated in PBS at 37°C for 3 weeks and the PBS solution containing released MAP‐RGD was collected at the predetermined time points.

The soft tissue occlusive ability of the MAP‐RGD coating was analyzed using a customized transwell insert, where the Ti‐mesh membrane was attached instead of the original polycarbonate‐based permeable membrane. MAP‐RGD was coated on the Ti‐mesh membrane in the insert, and the Ti‐mesh insert was put into a 24‐well cell culture plate (SPL Life Sciences, Pocheon, Korea). The bare Ti‐mesh insert was employed as a negative control. A total of 5 × 10^4^ NIH3T3 fibroblast cells were seeded on the surface of each Ti‐mesh insert and incubated in Dulbecco's modified Eagle's medium (DMEM; HyClone, Logan, UT, USA) supplemented with 10% (v/v) fetal bovine serum (FBS; HyClone) and 1% (v/v) penicillin–streptomycin (HyClone) at 37°C in a humidified incubator containing 5% CO_2_ and 95% air. After 24 h of incubation, the number of cells passaged across the membrane on the surface of the well plate was determined using a hemocytometer. Cell counts on the well plate surface at 1, 24, and 72 h postseeding were also quantified by measuring the absorbance at 450 nm using a microplate absorbance spectrometer (Perkin Elmer, Waltham, MA, USA) after treatment with the Cell Counting Kit reagent (CCK‐8; Dojindo, Tokyo, Japan).

### 4.3 Evaluation of in vitro osteogenic cellular behaviors

For in vitro analyses, a blended solution of 5 mg/ml MAP‐RGD and 50 μg/ml rhBMP‐2 was coated onto polystyrene surfaces (SPL Life Sciences) as mentioned above. Rat bone marrow mesenchymal stem cells (MSCs; ScienCell Research Laboratories, Carlsbad, CA, USA) were cultured and maintained in mesenchymal stem cell medium (ScienCell Research Laboratories) supplemented with 5% (v/v) FBS (ScienCell Research Laboratories), 1% (v/v) mesenchymal stem cell growth supplement (ScienCell Research Laboratories) and 1% (v/v) penicillin–streptomycin (ScienCell Research Laboratories) at 37°C in a humidified incubator containing 5% CO_2_ and 95% air. All assays were performed with cells in the passage range of 4–6, and the viability of cells was determined using a trypan blue (Sigma‐Aldrich) exclusion assay. Prior to each experiment, the sample surfaces were sterilized by soaking in excess Dulbecco's phosphate‐buffered saline (DPBS; HyClone) under UV radiation.

A total of 5 × 10^4^ cells (>95% viable) in serum‐free medium were seeded on each sample surface and then incubated for 1 h to allow adhesion onto the surface; then, the sample surfaces were extensively washed with DPBS to remove the unattached or loosely tethered cells. The attached cells on each surface were further cultured for 72 h to assess their proliferation levels. The number of cells at 1 h (for attachment analysis) and cell populations at every 24 h (for proliferation analysis) were quantified using the CCK‐8 assay.

Despite the osteoinductive ability, the treatment of BMP‐2 alone could be not sufficient to ensure the cell maturation of MSCs and subsequent matrix mineralization.^44^ The addition of ascorbic acid is crucial for MSC proliferation and collagen I fiber‐based extracellular matrix synthesis, and the sodium phosphate monobasic is known as a source of inorganic phosphate necessary for matrix mineralization.^45^, ^46^ After incubation to ~90% confluence, the MSCs on the sample surfaces were cultured in culture medium supplemented with 5 μg/ml ascorbic acid (Sigma‐Aldrich) and 1 mM monobasic sodium phosphate monobasic (Sigma‐Aldrich), which corresponds to 10% of the commonly used concentration for the induction of differentiation. For immunoblotting analysis, cells were lysed in radioimmunoprecipitation assay buffer (RIPA; Sigma‐Aldrich) supplemented with protease inhibitor cocktail (Sigma‐Aldrich) at 1 day after induction. The lysates were harvested by scrapping and were placed on ice for 30 min. After centrifugation at 10,000 × *g* for 15 min, the total content of soluble proteins in the supernatant was determined using the Bradford assay. Equivalent amounts of protein samples were boiled in Laemmlli buffer (Sigma‐Aldrich) and fractionated by 8% (v/v) SDS‐PAGE, followed by transfer to a nitrocellulose membrane (GE Healthcare, München, Germany). The membrane was treated with 5% (v/v) nonfat dry milk overnight at 4°C for blocking, and then exposed to antibodies against FAK (1:500; Abcam, Cambridge, UK), phosphorylated FAK (pY397‐FAK; 1:500; Abcam), BMPR‐IA (1:500; Abcam), BMPR‐IB (1:500; Abcam), BMPR‐II (1:1000; Abcam) and glyceraldehyde‐3‐phosphate dehydrogenase (GAPDH; 1:10,000; Abcam) for 1 h. The immunoreactive bands on the membrane were visualized colorimetrically with 5‐bromo‐4‐chloro‐30‐indolyphosphate p‐toluidine salt (BCIP; Roche, Basel, Switzerland) and nitroblue tetrazolium (NBT; Roche) after incubation with an ALP‐conjugated secondary antibody (1:30,000; Sigma‐Aldrich). The band intensities were quantified using CLIQS 1D Pro (TotalLab, Newcastle, UK) software with a loading control of GAPDH.

For the phenotypic analysis of osteogenic differentiation, the ALP activity of cells was determined at 4, 7, and 14 days after induction using the SensoLyte® pNPP Alkaline Phosphatase Assay Kit (Anaspec, Fermont, CA, USA) following the manufacturer's protocol. The deposition of calcium minerals in differentiated MSCs was also assessed using Alizarin Red S (adjusted to pH 4.2 with ammonium hydroxide; Sigma‐Aldrich) staining at 7, 14, and 21 days after induction. The stained cells were washed with DW three times and observed using an optical microscope (Olympus). The retained dye was eluted into 20% (v/v) acetic acid to quantify the deposited calcium mineral by determining the absorbance at 570 nm using a microplate absorbance spectrometer.

The genotypic analyses of osteogenic commitment were performed by quantifying the expression of transcription factors, including Runx2, OSX and ATF4, and osteogenic markers, including T1Col, ALP, BSP, and OCN. Total cellular RNA was isolated from the differentiated cells at 1, 4, 7, 14, 21, and 28 days after induction using TRIzol® reagent (Invitrogen, Carlsbad, CA, USA). The amount of purified RNA was determined by measuring the absorbance at 260 nm using a UV–Vis spectrophotometer (Mecasys, Seoul, Korea), and the RNA was reverse‐transcribed into cDNA using Topscript™ RT DryMix (dT18; Enzynomics, Daejeon, Korea). The obtained cDNA was subjected to 40 cycles of amplification with oligonucleotide primers for the target genes (Table S1, Supporting Information) via real‐time RT‐PCR using the StepOne Plus™ Real‐Time PCR System (Thermo Fisher, Waltham, MA, USA). The amplified genes were detected at a specific fluorescence intensity (Ct, cycle threshold) and the fold changes in their expression were determined with the reference gene GAPDH using the comparative Ct method.^47^

### 4.4 In vivo bone regeneration analyses

All animal experiments were performed in a facility with a specific‐pathogen‐free‐2 (SPF‐2) level, and the experimental protocol was approved by the Institutional Animal Care and Use Committee of the Pohang University of Science and Technology (POSTECH IACUC‐2015‐0003). Male Sprague–Dawley rats (7 weeks old, 250–300 g; Orient Bio, Seongnam, Korea) were housed one per cage with soft bedding with free access to food and water in a temperature (23°C) and humidity (50%)‐controlled environment (*n* ≥ 5). Prior to surgery, each rat was anesthetized via intramuscular injection of 10 mg/kg Rompun (xylazine; Bayer, Leverkusen, Germany) and 30 mg/kg Zoletile 50 (tiletamine and zolazepam; Virbac, Carros, France). The scalp and periosteum were carefully incised, and then a circular critical‐sized defect was generated by punching the exposed calvaria with an 8‐mm diameter trephine drill under continuous saline irrigation. The defect site was covered with each Ti‐mesh sample, and the sample surface was fastened with two fixing screws. Then, the incised scalp and periosteum were sealed in layers with 5–0 Vicryl sutures (Ethicon, Somerville, NJ, USA).

For radiographic and histological analyses, the rats were euthanized at 8 weeks after surgery, and the calvarial specimens were fixed with 10% (v/v) formalin (Sigma‐Aldrich) for 24 h. The radiographs of the specimens were taken using microcomputed tomography (μ‐CT; Inveon; Siemens, Knoxville, TN, USA). A total of 1024 slices were imaged for each specimen, and the 3D reconstructed images (1024 × 1024 pixels) were analyzed to determine the volume fraction of regenerative bone (BV/TV); the thickness of newly generated bone; several parameters of trabecular microarchitecture, including the Tb.Th, Tb.N, and Tb.Sp; and the bone mineral density (BMD) using Inveon Research Workplace software (Siemens).

The rat calvarial specimens were decalcified in a decal solution (Calci‐Clear Rapid; National Diagnostics, Somerville, NJ, USA) for 24 h, embedded in paraffin, and cut into 3 μm thick sections. The tissue sections were stained with MT (Sigma‐Aldrich) and H&E (Sigma‐Aldrich) after deparaffinization and hydration. Histology photographs were obtained under a BX‐41 microscope (Olympus) and analyzed using an image analysis program (CaseViewer v.2.0; 3DHISTECH, Budapest, Hungary). Histology was graded on a semiquantitative scale of 0 to 3 based on the quality of bone, defect bridging, vascularization, and inflammation (outlined in Table S2, Supporting Information).

### 4.5 Statistical analysis

In vitro experiments were performed independently at least in triplicate, and three samples were analyzed for each experiment. In vivo data were obtained from the animal surgery of at least five individual rats. The normality of the quantitative data distribution was assessed using the Shapiro–Wilk test. The significance of the data obtained from each group was statistically analyzed via one‐way ANOVA with Tukey's post hoc test (for a normal distribution) or Kruskal–Wallis test with Dunn's post hoc test (for a nonnormal distribution). The data are presented as the mean ± SD with statistical significance. The significance of the data for an experimental group is designated with the notation “a” (*p* < 0.05), “b” (*p* < 0.01), and “c” (*p* < 0.005). All data were processed by R software (v.3.2.1; R Development Core Team, Vienna, Austria).

## AUTHOR CONTRIBUTIONS

**Yun Kee Jo:** Conceptualization (equal); investigation (lead); methodology (lead); writing – original draft (lead); writing – review and editing (supporting). **Bong‐Hyuk Choi:** Conceptualization (supporting); investigation (supporting). **Cong Zhou:** Investigation (supporting). **Sang Ho Jun:** Conceptualization (equal); methodology (equal); supervision (equal). **Hyung Joon Cha:** Conceptualization (equal); supervision (equal); writing – review and editing (lead).

## FUNDING INFORMATION

This work was financially supported by the Korea Health Technology R&D Project through the Korea Health Industry Development Institute (KHIDI) funded by the Ministry of Health & Welfare, Republic of Korea (grant number: HI22C1347 to Hyung Joon Cha, Sang Ho Jun, and Yun Kee Jo), the High Value‐added Food Technology Development Program through the Korea Institute of Planning and Evaluation for Technology in Food, Agriculture and Forestry (IPET) funded by the Ministry of Agriculture, Food & Rural Affairs (MAFRA), Republic of Korea (grant number: 321025051HD060 to Hyung Joon Cha & Yun Kee Jo), and the BK21 Four Education Program for Innovative Chemical Engineering Leaders through the National Research Foundation of Korea (NRF) (to Hyung Joon Cha).

## CONFLICT OF INTEREST

The authors declare that they have no conflict of interest.

### PEER REVIEW

The peer review history for this article is available at https://publons.com/publon/10.1002/btm2.10493.

## Supporting information


**Table S1.** Primers used for gene expression analyses.
**Table S2**. Histological scoring matrix used to grade bone healing.
**Figure S1.** Degradation profile of different concentrations of MAP‐RGD coating for 3 weeks. The data are presented as the mean ± SD with statistical significance (Kruskal‐Wallis test with Dunn's post hoc test). *p* < 0.005 is noted as “c”. Statistical insignificance is noted as “ns.”
**Figure S2.** SEM images of the MAP‐RGD@BMP‐2‐coated surface at 1 day after seeding of fibroblasts. The white arrows indicate the holes formed by local degradation of MAP‐RGD coating.

## Data Availability

The data that support the findings of this study are available from the corresponding author upon reasonable request.
