# Supplementary material for: Cell recognitive bioadhesive‐based osteogenic barrier coating with localized delivery of bone morphogenetic protein‐2 for accelerated guided bone regeneration
Source: Bioeng Transl Med. 2023 Jan 18;8(3):e10493. doi: 10.1002/btm2.10493 (PMC10189428; doi:10.1002/btm2.10493)
Supplement: Supplementary file 1 — Table S1. Primers used for gene expression analyses. Table S2. Histological scoring matrix used to grade bone healing. Figure S1. Degradation profile of different concentrations of MAP‐RGD coating for 3 weeks. The data are presented as the mean ± SD with statistical significance (Kruskal‐Wallis test with Dunn's post hoc test). p < 0.005 is noted as “c”. Statistical insignificance is noted as “ns.” Figure S2. SEM images of the MAP‐RGD@BMP‐2‐coated surface at 1 day after seeding of fibroblasts. The white arrows indicate the holes formed by local degradation of MAP‐RGD coating. [file BTM2-8-e10493-s001.docx]

**Supporting Information**

**Cell recognitive bioadhesive-based osteogenic barrier coating with localized delivery of bone morphogenetic protein-2 for accelerated guided bone regeneration**

Yun Kee Jo^1,2^, Bong-Hyuk Choi^3^, Cong Zhou^4^, Sang Ho Jun^5^ and Hyung Joon Cha^6^

^1^Department of Biomedical Convergence Science and Technology, School of Convergence, Kyungpook National University, Daegu 41566, Republic of Korea

^2^Cell and Matrix Research Institute, Kyungpook National University, Daegu 41944, Republic of Korea

^3^Nature Gluetech Co., Ltd, Seoul 08502, Republic of Korea

^4^School of Stomatology, Shandong University, Jinan 17923, China

^5^Department of Oral and Maxillofacial Surgery, Korea University Anam Hospital, Seoul 02841, Republic of Korea

^6^Department of Chemical Engineering, Pohang University of Science and Technology, Pohang 37673, Republic of Korea

*Correspondence to: hjcha@postech.ac.kr (H. J. Cha), junsang@korea.ac.kr (S. H. Jun)

**Table S1.** Primers used for gene expression analyses.

| **Marker gene** | **Primer sequences (5’→3’)** | | **Accession No.** |
| --- | --- | --- | --- |
| GAPDH | Forward | TTGTCTCCTGCGACTTCAACA | NM_001289726.1 |
|  | Reverse | GTGGTCCAGGGTTTCTTACTCC |  |
| Runx2 | Forward | GACGTGCCCAGGCGTATTTC | NM_001146038.2 |
|  | Reverse | AAGTCTGGGGTCCGTCAAGG |  |
| OSX | Forward | GAGGCACAAAGAAGCCATACACT | NM_181374 |
|  | Reverse | AGTCCATTGGTGCTTGAGAAGG |  |
| ATF4 | Forward | GACCGAGATGAGCTTCCTGAACAG | NM_024403.2 |
|  | Reverse | CCGCCTTGTCGCTGGAGAAC |  |
| T1Col | Forward | GAGCGGAGAGTACTGGATCG | BC050014 |
|  | Reverse | GCTTCTTTTCCTTGGGGTTC |  |
| ALP | Forward | GCCCTCCAGATCCTGACCAA | NM_007431.3. |
|  | Reverse | GCAGAGCCTGCTTGGCCTTA |  |
| BSP | Forward | TCCATCGAAGAATCAAAGCA | L20232.1 |
|  | Reverse | ATGAGCGTGGCCGGTACTTA |  |
| OCN | Forward | AGGAGGGCAATAAGGTAGTGAA | NM_007541.3 |
|  | Reverse | TACCATAGATGCGTTTGTAGGC |  |

**Table S2**. Histological scoring matrix used to grade bone healing.

| **Score** | **Quality of new bone** | **Defect bridged by new bone formation** | **Vascularization** | **Inflammation** |
| --- | --- | --- | --- | --- |
| 0 | No bone | None | None | Severe |
| 1 | Predominantly woven bone | Little stumps | Present at the periphery | Moderate |
| 2 | Mixed woven and lamellar bone | Moderate with small gaps | Present centrally | Mild |
| 3 | Predominantly lamellar bone | Complete | Abudant at the periphery and centrally | None |

**
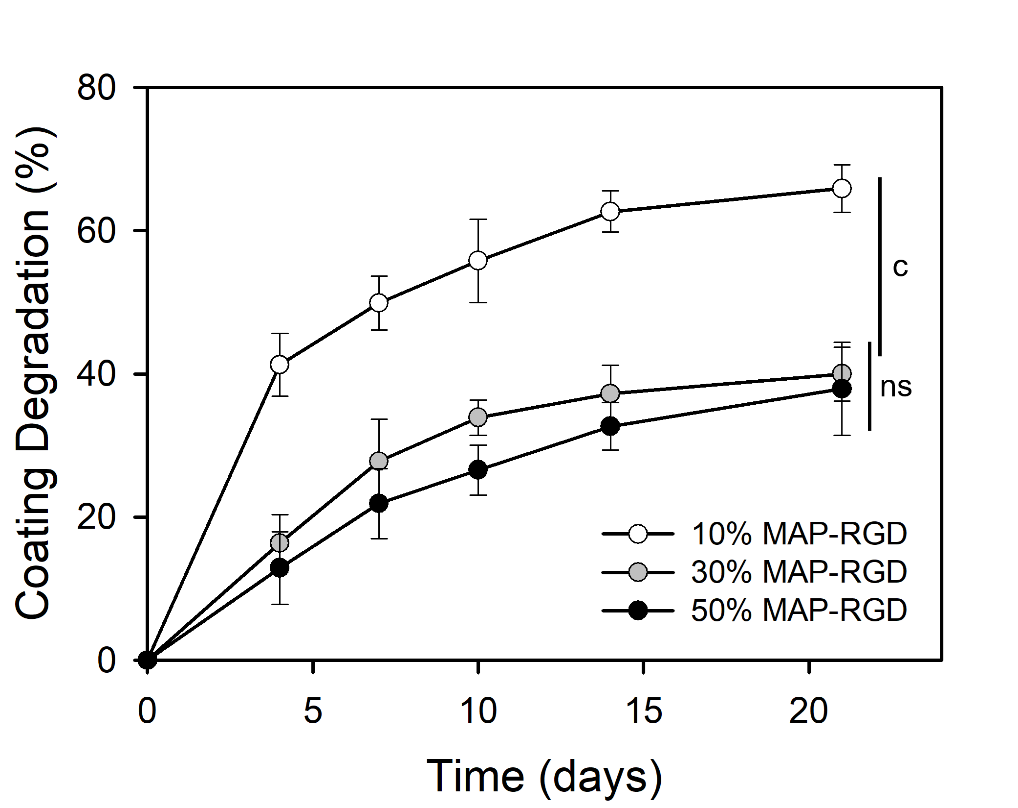
**

**Figure S1.** Degradation profile of different concentrations of MAP-RGD coating for 3 weeks. The data are presented as the mean ± standard deviation with statistical significance (Kruskal-Wallis test with Dunn’s post-hoc test). *p*<0.005 is noted as “c”. Statistical insignificance is noted as “ns”.


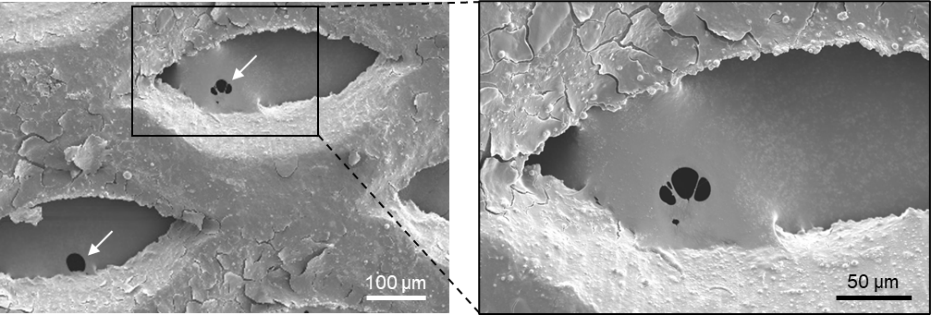


**Figure S2.** SEM images of the MAP-RGD@BMP-2-coated surface at 1 day after seeding of fibroblasts. The white arrows indicate the holes formed by local degradation of MAP-RGD coating.
